# An open-label study on the short-term effects of a novel EFSA-compliant nutraceutical combination in mild-to-moderate hypercholesterolemia

**DOI:** 10.22038/AJP.2022.20662

**Published:** 2022

**Authors:** Piercarlo Minoretti, Marco Biagi, Enzo Emanuele

**Affiliations:** 1 *Studio Minoretti, Oggiono (LC), Italy*; 2 *Department of Environment, Earth and Physical Sciences, University of Siena, Siena, Italy*; 3 *2E Science, Robbio (PV), Italy*

**Keywords:** Monacolins, Berberine, Pomegranate, Nutraceutical, Hypercholesterolemia, Primary prevention

## Abstract

**Objective:**

Recently, the European Food Safety Authority (EFSA) has recommended to limit the use of total monacolins in red yeast rice (RYR) products to a dose <3 mg/day. However, data concerning the lipid lowering efficacy of the reduced dosage remain limited. A monacolin dose reduced due to safety issues may be expected to be less effective as a lipid lowering strategy and, for this reason, nutraceutical combinations with other active compounds may offer a viable solution as they can act synergistically through different mechanisms.

**Materials and Methods:**

This 8-week open-label study was designed to investigate the safety and efficacy of a novel ESFA-compliant lipid lowering nutraceutical combination (Colestarmony Plus^®^; containing total monacolins from RYR at a dose of 2.9 mg/day, a highly bioavailable berberine formulation, and pomegranate extract) in subjects (n=40) with mild-to-moderate hypercholesterolemia and no history of cardiovascular disease.

**Results:**

After 8 weeks of supplementation, Colestarmony Plus^® ^significantly reduced total cholesterol (−10.4%, p<0.05), low-density lipoprotein cholesterol (−14.8%, p<0.05), oxidized low-density lipoprotein cholesterol (−12.0%, p<0.05), and high-sensitivity C-reactive protein (−14.0%, p<0.05) compared with baseline values. A subgroup of 22 patients underwent measurements of flow-mediated dilation, with values increasing by 18.0% at 8 weeks with respect to baseline (p<0.05). The supplement was generally well-tolerated.

**Conclusion:**

Our short-term results indicate that the tested ESFA-compliant nutraceutical is effective in a primary prevention setting, even by providing only <3 mg/day of monacolins.

## Introduction

The use of 3-hydroxy-3-methylglutaryl–coenzyme A (HMG-CoA) reductase inhibitors (statins) is a mainstay of cardiovascular disease (CVD) prevention that acts by lowering cholesterol (Chou et al., 2016 ▶). While being among the most commonly used prescription drugs worldwide, statins are discontinued by nearly a third of patients without medical advice because of perceived adverse effects – including muscle-related symptom, weakness, and fatigue (Brown and Watson, 2018 ▶). In this scenario, the risk-benefit ratio of these drugs for primary prevention of CVD in otherwise healthy people with mildly to moderately increased cholesterol concentrations, remains controversial (Kazi et al., 2017 ▶). In an effort to avoid statin-related safety concerns, lipid-lowering nutraceuticals have extensively been prescribed in real world practice as a potential alternative to statins as a primary prevention strategy (Poli et al. 2018 ▶). While several components from herbal products have shown cholesterol-lowering efficacy via different mechanisms of action (Dougnon et al., 2014 ▶; Ghannadi et al., 2015 ▶; Alaei et al, 2020 ▶), red yeast rice (RYR) has been the most extensively investigated (Banach et al., 2019 ▶). The fermentation process of RYR by *Monascus purpureus *leads to the production of pharmacologically active lipid-lowering ingredients – including monacolin K, which is structurally identical to the prescription drug lovastatin (Xiong et al., 2019 ▶). As a consequence, RYR nutraceuticals containing monacolin K have a potential for producing adverse effects similar to that expected for pharmacological HMG-CoA inhibitors (Gerard et al., 2015 ▶). This has recently prompted the European Food Safety Authority (EFSA; *Draft regulation SANTE/10408/2020*) to maintain that the current use levels of monacolin K from RYR (10 mg/day) can raise safety concerns. Therefore, the EFSA recommended to limit the use of total monacolins from RYR to a dose of <3 mg/day. 

While there is an ample literature on the cholesterol-lowering efficacy of the currently unauthorized monacolin K dosage (10 mg/day) (Cicero et al., 2019 ▶), data concerning the effectiveness of the approved reduced dose (<3 mg/day of total monacolins) remain limited. A lower monacolin dosage due to safety issues may be expected to be less effective as a lipid lowering strategy and, for this reason, nutraceutical combinations with other active compounds may offer a viable solution, as they can act synergistically through different mechanisms. Berberine, a natural isoquinoline alkaloid, can lower lipid levels by reducing the hepatic cholesterol synthesis and increasing LDL receptor expression (Fatahian et al., 2020 ▶). Unfortunately, berberine is characterized by low oral bioavailability and extensive first-pass drug metabolism (Liu et al., 2016 ▶). In this context, strategies to increase its absorption rate (including liposomal formulation) may improve its antilipidemic effects (Allijn et al., 2017 ▶). Growing evidence also indicates that pomegranate extract can reduce dyslipidemia and exert significant antioxidant and anti-inflammatory effects (Wang et al., 2018 ▶).

This 8-week open-label study was designed to investigate the safety and efficacy of a novel ESFA-compliant lipid lowering nutraceutical combination (Colestarmony Plus^®^; containing total monacolins from RYR at a dose of 2.9 mg/day, a highly bioavailable berberine formulation, and pomegranate extract) in subjects (n=40) with mild-to-moderate hypercholesterolemia and no history of cardiovascular disease. 

## Materials and Methods

**Design**

This was a single-blind, open-label study of a new combined ESFA-compliant cholesterol-lowering nutraceutical (Colestarmony Plus^®^; Biodue SpA, Barberino Tavarnelle, Italy) that contained RYR (59 mg, providing total monacolins at a dose of 2.9 mg/day), a liposomal formulation of berberine (500 mg), and pomegranate extract (50 mg). The supplement was given as tablets (1.1 g). All participants – who had mildly to moderately increased cholesterol concentrations and a negative history of cardiovascular disease – were recruited within a cardiovascular prevention program, as previously described (Biagi et al., 2018 ▶). The subjects were advised to keep their usual dietary patterns and levels of physical activity throughout the study. The study was approved by the local ethics committee (approval number E06/21) and written informed consent was obtained from all participants.

**Study participants**

Inclusion criteria were as follows: 1) age >18 years; 2) presence of mild-to-moderate hypercholesterolemia, defined as LDL-C levels between 115 and 180 mg/dl; total cholesterol (TC) levels between 200 and 260 mg/dl, and triglyceride (Tg) levels <250 mg/dl (Biagi et al., 2018 ▶; D’Addato et al., 2017 ▶); and 3) ability to provide written informed consent. The following exclusion criteria were applied: known personal or family history of CVD; use of drugs or food supplements with lipid lowering effects in the two months before enrollment; liver or muscle disease; familial hypercholesterolemia; intolerance to supplement components; breastfeeding; or pregnancy. 

**Procedures**

The study duration was 8 weeks. At baseline, all subjects (n=40; 21 men and 19 women) underwent a physical exam that included blood pressure measurements and quantification of the body mass index (BMI). All participants self-administered the nutraceutical once per day (one tablet after dinner). 

**Endpoints**

The primary outcome measures were the changes in lipid parameters before and after 8 weeks of supplementation. The modifications of inflammatory parameters served as secondary endpoints. Data regarding flow-mediated dilation (FMD) before and after 8 weeks of supplementation were available for 22 subjects (12 men and 10 women) and were included in an exploratory analysis.

**Biochemical analyses**

Lipid parameters were measured on a Hitachi-912 Auto Analyzer (Hitachi, Mannheim, Germany). Oxidized low-density lipoprotein (oxLDL) and high-sensitivity C-reactive protein (hs-CRP) levels were quantified with a commercially available immunoassay (Mercodia AB, Uppsala, Sweden) and an immunonephelometric assay (Dade Behring, Newark, DE, USA), respectively. Intra-assay and interassay coefficients of variation (CsV) for the hs-CRP assay were 6.7% and 3.8%, respectively. Tumor necrosis factor (TNF)-α concentrations were determined with an immunoassay (Titer-Zyme EIA kit; Assay Designs, Ann Arbor, MI, USA). Intra-assay and interassay CsV were 7.6% and 5.1%, respectively.

**Flow-mediated dilation**

Measurements of FMD (n=22) at baseline and after 8 weeks of supplementation were carried out in an outpatient setting. All subjects were in a fasting state. FMD was measured in the longitudinal plane at the right brachial artery. All measurements were performed with an 8.8-MHz linear array transducer positioned above the antecubital fossa, according to international recommendations (Thijssen et al., 2019 ▶).

**Safety assessment**

Safety was assessed using laboratory tests and collection of clinical adverse events (gastric pain, nausea, and muscle pain). The following definitions for clinically significant laboratory abnormalities were used: hepatic transaminases ≥3 × upper limit of normal (ULN), hematocrit decline ≥5% from baseline, hemoglobin decline ≥2 g/dl from baseline; creatinine ≥1.3 × ULN, and blood urea nitrogen (BUN) ≥2 × ULN (Nannoni et al., 2020 ▶). All safety laboratory testing was performed on an Auto Analyzer (Hitachi).

**Statistical analysis**

The study had an exploratory nature and no formal sample size estimation was undertaken. Continuous variables are expressed as means±standard deviations and were tested with the paired Student’s *t*-test for intragroup differences over time. Skewed variables were log-transformed before data analysis to improve normality. SPSS 20.0 (IBM, Armonk, NY, USA) was used for data analysis and two-tailed P values <0.05 were considered statistically significant; therefore, 95% confidence intervals (CIs) were estimated for differences from baseline to 8 weeks for primary and secondary endpoints.

## Results

The study sample consisted of 40 subjects who had mildly to moderately increased cholesterol concentrations and a negative history of cardiovascular disease (Table 1). Safety measures at baseline – including hepatic transaminases, hematocrit, hemoglobin, creatinine, and BUN – were within their reference ranges (data not shown). 

**Table 1:** General characteristics of the study participants

Variable	Value* (n=40)
Men/women	21/19
Age, years	46.9±7.1
Body mass index, kg/m^2^	25.8±2.3
Systolic blood pressure, mm Hg	125±9
Diastolic blood pressure, mm Hg	79±7
Fasting plasma glucose, mg/dl	91.7±7.9

∗Data are given as counts for sex, whereas all other variables are summarized as means±standard deviations.

Table 2 shows the differences and 95% CIs observed from baseline to 8 weeks for primary and secondary endpoints. After 8 weeks of supplementation, significant reductions were observed compared with baseline values for plasma TC (−10.4%, p<0.05), LDL-C (−14.8%, p<0.05), oxLDL (−12.0%, p<0.05), and hs-CRP (−14.0%, p<0.05). No relevant effects were identified with respect to HLD-C, Tg, or TNF-α levels. A subgroup of 22 patients underwent measurements of FMD, with values increasing by 18.0% (from 7.2±3.8% to 8.5±3.4%, p<0.05). In general, the supplement was well-tolerated. Two subjects (4%) had gastric pain but no discontinuation was required. No study participant developed nausea or muscle pain. Additionally, no clinically significant laboratory abnormalities were observed from baseline to 8 weeks.

**Table 2:** Variations of lipid profile and inflammatory markers after 8 weeks of supplementation with a novel EFSA-compliant nutraceutical combination in 40 subjects with mild-to-moderate hypercholesterolemia and no history of cardiovascular disease

Biochemical variable	Baseline	8 weeks	Δ 8 weeks-baseline (95% CI)
TC, mg/dl	230±17	206±14*	-24 (-31; -19)
LDL-C, mg/dl	142±18	121±13*	-21 (-29; -16)
OxLDL, U/L	50±14	43±12*	-7 (-13; -3)
HDL-C, mg/dl	47±8	49±7	+2 (-2; +6)
Tg, mg/dl	139±47	131±45	-8 (-17; +12)
Hs-CRP, mg/L	1.4±0.8	1.2±0.5*	-0.2 (-0.4; -0.1)
TNF-α, ng/ml	1.6±0.8	1.6±0.6	0 (-0.2; +0.2)

Data are expressed as means and standard deviations. *p<0.05 *versus *baseline (Student’s *t*-test for paired data). Abbreviations: CI, confidence interval; TC, total cholesterol; LDL-C, low-density lipoprotein cholesterol; OxLDL, oxidized low density lipoprotein; HDL-C, high-density lipoprotein cholesterol; Tg, triglyceride; Hs-CRP, high-sensitivity c-reactive protein; TNF, tumor necrosis factor.

## Discussion

Findings from previous studies demonstrated that 10 mg of monacolin K from RYR – either alone or in combination with berberine – can exert significant lipid lowering effects with a reduction in LDL-C that ranged between 20% and 35% (Banach et al., 2019 ▶). As the EFSA recently recommended to limit the use of total monacolins from RYR to a dose <3 mg/day, a pilot study on the effects of the novel reduced dosage on lipid parameters, inflammatory markers, and endothelial function was required. Our preliminary results indicate that the novel EFSA-compliant nutraceutical combination containing total monacolins from RYR at a dose of 2.9 mg/day, a highly bioavailable berberine formulation, and pomegranate extract effectively reduced TC and LDL-C in subjects who had mildly to moderately increased cholesterol concentrations. However, the magnitude of LDL-C decrease following 8 weeks of supplementation, was less prominent (−14.8%) than that previously reported for 10 mg of monacolin K (20–35%) (Banach et al., 2019 ▶; Poli et al., 2018 ▶). A decrease of similar extent was observed for TC (−10.4%), hs-CRP (−14.0%), and oxLDL (−12.0%). Collectively, these results suggest that the cholesterol lowering, anti-inflammatory, and antioxidant effects elicited by the nutraceutical combination containing 2.9 mg/day of total monacolins were not as prominent as those obtained with the previous standard usage level (10 mg/day of monacolin K). While this can put into question whether the reduced dosage will be clinically meaningful for primary prevention of CVD, one interesting observation in our study is that 8 weeks of supplementation with the novel EFSA-compliant nutraceutical combination increased FMD values by 18.0% in a subgroup of 22 study participants. FMD obtained at the brachial artery is a non-invasive method of endothelial function assessment (Thijssen et al., 2018 ▶). While these results suggest that the tested nutraceutical combination has the potential to maintain healthy circulatory function, it should be noted that the all participants who underwent FMD measurements, had normal values at baseline. These findings warrant further research on this supplement in patients with lower baseline FMD due to the presence of overt CVD or a higher burden of risk factors. The tested FSA-compliant nutraceutical combination was well-tolerated, and no cases of muscle pain or nausea were observed in our study. Although gastric pain occurred in two participants, none of them discontinued taking the supplement. 

This is, to our knowledge, the first study to examine the effects of the novel EFSA-compliant nutraceutical combination containing total monacolins from red yeast rice at a low dose (<3 mg/day) in a primary prevention setting. However, several limitations must be considered. First, the open-label nature of the design without a placebo arm could be associated with observer bias. Second, the sample size was limited and larger investigations are required to confirm and expand our findings. Finally, it would have been interesting to include patients with overt CVD and to evaluate the role of the supplement in a secondary prevention framework.

In summary, our short-term results indicate that the tested ESFA-compliant nutraceutical is safe and effective in a primary CVD prevention setting, even if providing only 2.9 mg/day of total monacolins. Significant reductions in TC, oxLDL, and hs-CRP were notably accompanied by an increase in FMD. 

## Conflicts of interest

Enzo Emanuele is the unique owner of 2E Science (Robbio, Italy), a privately held research organization that partially funded this study.
